# Presence of galactose in precultures induces *lacS* and leads to short lag phase in lactose-grown *Lactococcus lactis* cultures

**DOI:** 10.1007/s10295-018-2099-0

**Published:** 2018-11-09

**Authors:** Bettina Lorántfy, Anna Johanson, Fábio Faria-Oliveira, Carl Johan Franzén, Valeria Mapelli, Lisbeth Olsson

**Affiliations:** 1 Division of Industrial Biotechnology, Department of Biology and Biological Engineering, Chalmers University of Technology, 412 96 Gothenburg, Sweden; 2 Chr. Hansen A/S, 2970 Hørsholm, Denmark

**Keywords:** *Lactococcus lactis*, Lag phase, Galactose–lactose antiporter, Starter cultures, Galactose

## Abstract

Lactose conversion by lactic acid bacteria is of high industrial relevance and consistent starter culture quality is of outmost importance. We observed that *Lactococcus lactis* using the high-affinity lactose-phosphotransferase system excreted galactose towards the end of the lactose consumption phase. The excreted galactose was re-consumed after lactose depletion. The *lacS* gene, known to encode a lactose permease with affinity for galactose, a putative galactose–lactose antiporter, was upregulated under the conditions studied. When transferring cells from anaerobic to respiration-permissive conditions, lactose-assimilating strains exhibited a long and non-reproducible lag phase. Through systematic preculture experiments, the presence of galactose in the precultures was correlated to short and reproducible lag phases in respiration-permissive main cultivations. For starter culture production, the presence of galactose during propagation of dairy strains can provide a physiological marker for short culture lag phase in lactose-grown cultures.

**Electronic supplementary material:**

The online version of this article (10.1007/s10295-018-2099-0) contains supplementary material, which is available to authorized users.

## Introduction

Lactic acid bacteria (LAB) have a long history of use in a variety of fermented dairy products, and have usually been applied under anaerobic or microaerophilic conditions. *Lactococcus lactis* is a LAB used for the production of both fermented products and starter cultures and has been studied thoroughly under anaerobic conditions. Some *L. lactis* strains are aerotolerant, i.e., are able to grow aerobically using the coupled NADH oxidase/NADH peroxidase system, resulting in different end products from those produced under anaerobic growth conditions [26]. It has also been shown that *L. lactis* is able to respire when exogenous hemin is supplied to the growth medium [15] (here referred to as respiration-permissive condition). Hemin completes the electron transport chain for respiration, which is otherwise defective in *L. lactis*. Certain heme biosynthesis genes are missing from LAB, while the heme-dependent *bd*-type cytochrome is encoded by the *cydABCD* operon [6]. Respiratory metabolism decreases oxidative damage by lowering the intracellular oxygen concentration [34].

**Fig. 1:**
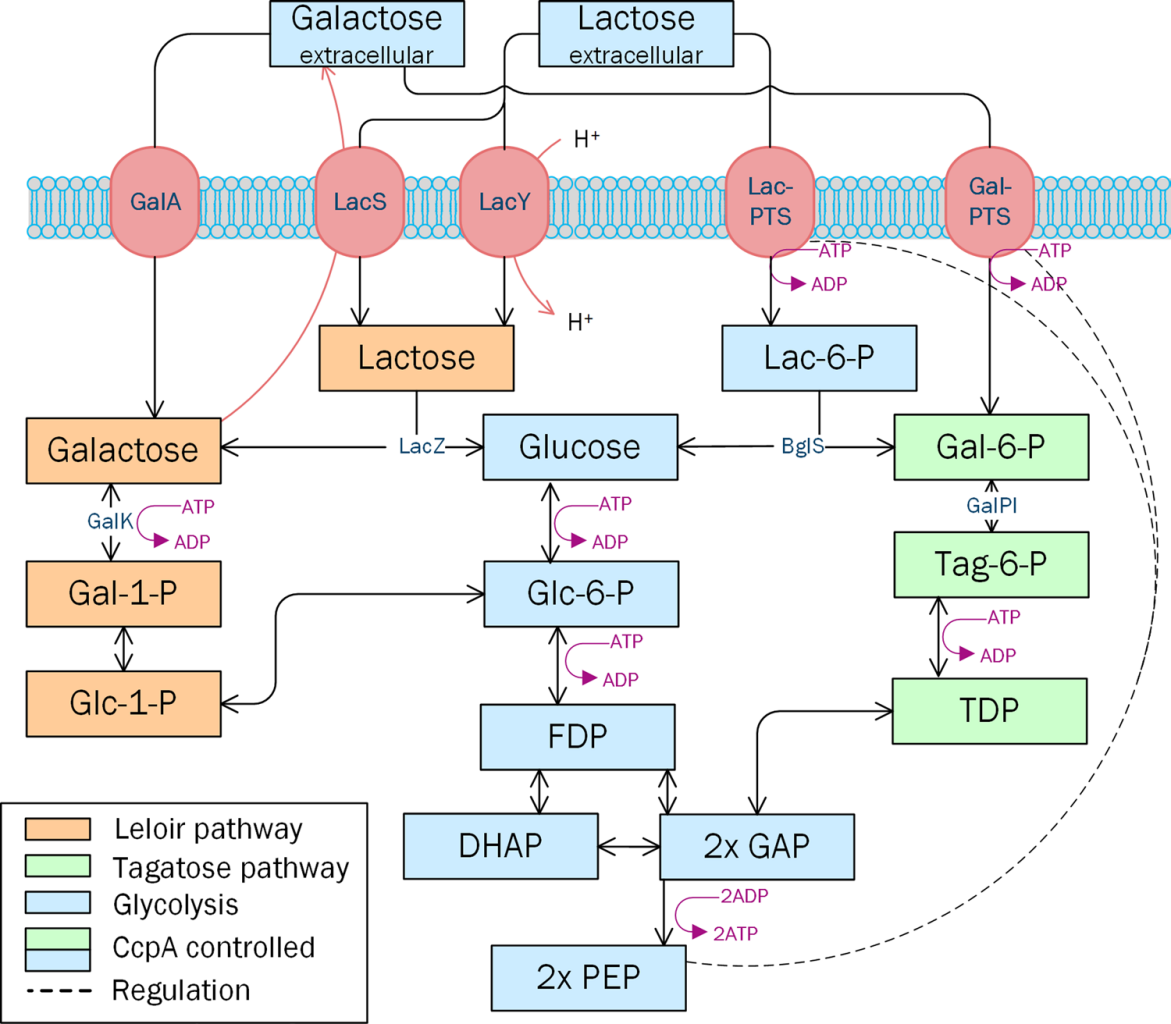
Uptake and partial metabolism of lactose and galactose in *L. lactis*. Lactose assimilation by the lactose permeases with the subsequent Leloir pathway (shaded orange), or by the PEP-dependent lac-PTS with the following tagatose pathway (shaded green). Galactose is taken up either by the galactose permease or the gal-PTS. Lactose and glucose catabolite repression in LAB is a tightly regulated mechanism by the catabolite control protein (CcpA), while galactose is a sugar that does not induce CcpA catabolic repression. The ATP and ADP requirements of each step are indicated. *GalA* galactose permease, *LacS* galactose–lactose antiporter, *LacY* lactose-proton symporter, *Lac-PTS* lactose-phosphotransferase system, *Gal-PTS* galactose phosphotransferase system, *Gal-1-P* galactose 1-phosphate, *Glc-1-P* glucose 1-phosphate, *Glc-6-P* glucose 6-phosphate, *F-6-P* fructose 6-phosphate, *FDP* fructose 1,6-bisphosphate, *DHAP* dihydroxyacetone phosphate, *GAP* glyceraldehyde 3-phosphate, *PEP* phosphoenolpyruvate, *GalK* galactokinase, *LacZ* β-galactosidase, *BglS* phospho-β-galactosidase, *GalPI* galactose-6-phosphate isomerase

LAB have evolved different systems for the uptake of lactose and its constituent monosaccharides, glucose and galactose [18]. The lactose-specific phosphoenolpyruvate (PEP)-dependent lactose-phosphotransferase system (lac-PTS) is the most bioenergetically efficient transport system for rapid lactose uptake. When lactose is taken up by lac-PTS, lactose uptake and phosphorylation are concomitant [33], and the lactose is metabolized as lactose-6 phosphate via the tagatose pathway and glycolysis (Fig. 1) [10].

While lactose uptake by lac-PTS is driven by the ∆G of the conversion of PEP into pyruvate, alternative permease transport systems, such as symports, antiports or uniports (Fig. 1) use concentration gradients for sugar uptake. Intracellular lactose is instead hydrolyzed to glucose and galactose by β-galactosidase, after which galactose is metabolized via the Leloir pathway (Fig. 1) [16]. It has also been reported that after prolonged incubation some lac-PTS plasmid-cured strains, which have lost the ability to assimilate lactose by the lac-PTS, assimilate lactose slowly by the permease system [8].

It is, however, not yet known which sugar transport systems are responsible for maintaining rapid lactose utilization when both lac-PTS and permease transporters are present. A galactose–lactose antiporter system has been described in lac-PTS negative *S. thermophilus* [17, 20] to facilitate lactose uptake by concomitant excretion of intracellular galactose. LacS lactose permease in *L. lactis* IL1403 was described as a putative H^+^-lactose symporter or galactose–lactose antiporter [4] with affinity for galactose [1, 37], but its role in sugar uptake has never been confirmed. In the absence of lactose, galactose uptake occurs via the galactose-PTS (gal-PTS) [29] or the galactose permease (*galA* also designated as *galP*) systems (Fig. 1) [16, 18]. If galactose is assimilated by the gal-PTS, it is concomitantly phosphorylated to galactose-6-phosphate, while in the case of galactose permease, galactose is assimilated and subsequently phosphorylated to galactose-1-phosphate (Fig. 1). For galactose uptake, some studies have been published on LAB physiology under anaerobic conditions [16, 36], but little information is available on the sugar transporters used for galactose in LAB and on galactose physiology under aerobic conditions [35].

The present study aimed at unravelling the impact of the presence of galactose in precultures on the lag phase of lactose-grown *L. lactis* main cultivations. We initially observed that galactose was excreted during lactose consumption by lac-PTS-positive *L. lactis* subsp. *lactis* cultures. The metabolism of galactose, excreted or supplemented was characterized under all cultivation conditions studied. Analysis of specific sugar uptake rates, β-galactosidase enzymatic activities, metabolic pathways, and *lacS* expression studies were used to shed light on the role of LacS in galactose excretion in *L. lactis* subsp. *lactis.* Our results indicate that the presence of galactose in the culture broth can be used as a marker to indicate when to harvest to achieve good performance in subsequent cultures.

## Materials and methods

### Strain, static flask growth conditions, and glycerol stock preparation

The lac-PTS-positive *Lactococcus lactis* subsp. *lactis* CHCC2862 was obtained from Chr. Hansen (Hørsholm, Denmark). Static flask cultivations on lactose were generally used as a preculture step. 250 mL flasks with 100 mL M17 medium (Oxoid, Thermo Fisher Scientific, Waltham, MA, USA) supplemented with 20 mM lactose (VWR, Radnor, PA, USA) were incubated at 30 °C and at starting pH of 6.5. Anaerobic conditions were conducted by keeping the flasks static and equipping them with rubber stoppers fitted with anaerobic fermentation loops containing glycerol plugs to protect the culture from outer air. The flask cultivation conditions were not strictly anaerobic; however, the extracellular metabolites, i.e., high lactic acid production and no acetoin production (data not shown) confirmed the anaerobic nature of the cultivations. Frozen glycerol stocks were prepared by harvesting cells at the late growth phase before lactose depletion, mixed with glycerol solution for a final glycerol concentration of 9% (V/V), and stored at − 80 °C. These frozen stocks were used to inoculate static flasks as well as bioreactor precultures.

Aerobic and respiration-permissive galactose cultures in 250 mL flasks with 100 mL M17 medium (Oxoid, Thermo Fisher Scientific, Waltham, MA, USA) supplemented with 28 mM galactose were inoculated at an initial OD_600_ of 0.1–0.2 and were incubated at 30 °C with 150 rpm agitation. For respiration-permissive cultivations, filter-sterilized hemin stock solution (Bovine hemin, ≥ 90% purity, Sigma-Aldrich, St. Louis, MI, USA) was added to a final concentration of 7.7 µM hemin. Cultures were carried out in biological duplicates. Off-line sampling was carried out every hour.

### Bioreactor cultivations: general cultivation conditions

Bioreactor cultivations were carried out on M17 medium (Oxoid, Thermo Fisher Scientific, Waltham, MA, USA) supplemented either with 20 mM lactose or 28 mM galactose in controlled stirred bioreactor vessels (DASGIP fermenter system with 8 parallel SR0700ODLS vessels, Eppendorf, Hamburg, Germany) at 30 °C. pH was controlled at 6.0 with 2 M NaOH. Bioreactor cultures were inoculated with an initial OD_600_ of 0.1–0.2 in a total volume of 500 mL. All reactors were equipped with pH and DO probes (Mettler Toledo probes, Mettler Toledo, Greifensee, Switzerland) and off-gas analyzers. Off-line analyses, off-gas rates, rates and yield calculations were carried out as described at Text S1.

At aerobic and respiration-permissive cultivations the dissolved oxygen control was set at a minimum threshold of 60% with a cascade control by gradually increasing the stirrer speed and the aeration rate using DASGIP control software (Eppendorf, Hamburg, Germany). The initial aeration rate was set to 0.4 vvm, with stirring at 400 rpm. The CO_2_ (%) and O_2_ (%) in the off-gas were monitored continuously using BlueSens gas analyzers (BlueSens Gas Technology GmbH, Herten, Germany) to calculate the volumetric CO_2_ evolution rate (CER, mmol L^−1^ h^−1^) and the O_2_ uptake rate (OUR, mmol L^−1^ h^−1^) (Text S1). For respiration-permissive cultivations, filter-sterilized hemin stock solution (Bovine hemin, ≥ 90% purity, Sigma Aldrich, St. Louis, MI, USA) was added to a final concentration of 7.7 µM hemin.

Anaerobic cultivations in the bioreactors were achieved by not gassing the reactors with 200 rpm stirrer speed. It was expected that as soon as the culture started to grow the minor remaining amounts of oxygen leave the reactor together with the produced carbon dioxide.

Anaerobic precultures in bioreactors were cultured under three different conditions: no pH control with monitoring only, on 20 mM lactose (Lac); pH control at pH 6.0, on 20 mM lactose and 9 mM galactose (Lac + Gal + pH); and pH control at pH 6.0, on 20 mM lactose (Lac + pH). Off-line sampling was carried out every 30 min. Cells were harvested at different time points and used as inoculum for the main cultivations in bioreactors applied with aeration and hemin supplementation. The main cultivations were inoculated with different preculture volumes to achieve an initial desired optical density.

The length of lag phase of the sequential batch culture was calculated using the first derivative of the CO_2_ (%) off-gas signals (Text S2). The end of the lag phase was determined as the time at which the first derivative had a non-zero value. The high frequency of data collection (CO_2_ recordings every min) allowed the length of the lag phase to be determined with an accuracy of 0.1 h. Each type of preculture was carried out in biological duplicates and the subsequent main cultivations were inoculated in two or three technical replicates (inoculated by the same precultures).

### β-galactosidase assay

The *p*-nitrophenol (pNP, Sigma Aldrich, St. Louis, MI, USA) assay was used to investigate the presence of extracellular β-galactosidases in the filtered culture medium [2]. Filtered culture broth (100 µL) was mixed with 50 µL 1 mM pNP substrate solution and 50 µL pH 6.0 P_i_ buffer. Samples were incubated at 30 °C in a water bath (Memmert, Schwabach, Germany) for 1 h and overnight. After incubation, 200 µL 0.2 M Na_2_CO_3_ was added to stop the reaction. The absorbance was read at 410 nm (Genesis 20 photometer, Thermo Scientific, Waltham, MA, USA). No difference was seen between the samples incubated for 1 h and overnight. Measurements were performed on blanks without the pNP substrate, and also on freshly prepared M17 medium (i.e., without the enzyme). Positive controls, consisting of 100 × diluted β-galactosidase (Megazyme β-galactosidase highly purified from *Aspergillus niger* in 3.2 M (NH_4_)_2_SO_4_, ~ 4000 U mL^−1^, Megazyme Inc. Chicago, IL, USA), were also analyzed. The absorption results were evaluated taking possible interfering compounds such as lactate, ethanol, and acetate into account (based on a personal communication with Thermo Scientific Protein Biology Technical Support and BioVision Inc. Technical Support, which are the two major providers of commercial pNP assays).

### RNA extraction, RNA quality and quantity, cDNA synthesis, primer design, qPCR and validation

RNase-free consumables and a clean working environment were used for RNA extraction. Triplicate samples were obtained from bioreactor batch cultures for RNA extraction. The volumes varied to ensure a similar range of total biomass for the RNA extraction step. Samples from batch cultures supplemented with galactose were collected at the time of the respiratory switch (Text S3), when hemin intake for respiration is relevant [21]. Samples from aerobic batch cultures supplemented with galactose were collected at the same time as the samples from respiration-permissive cultures, to ensure a similar range of biomass concentration. Samples from anaerobic preculture bioreactor batches were harvested at different times (reasoning is given in the Results section). RNA was extracted with Qiagen RNeasy Mini kit spin columns together with the Qiagen RNase-Free DNase Set for on-column DNA digestion (Qiagen N.V., Venlo, The Netherlands). Due to the high DNA load in the RNA samples, the instructions from the manufacturer were slightly modified (Text S3). The amount of RNA (µg mL^−1^) was measured along with the 260/280 and 260/230 absorbance ratio purity values (NanoDrop 2000, Thermo Scientific, Waltham, MA, USA), and RNA integrity was checked on conventional 1.5% agarose gel. cDNA was synthesized in duplicate 20 µL reaction mixtures with 2 µg total RNA (RT +) or without RNA (RT −) using the High-Capacity RNA-to-cDNA™ Kit with random hexamer priming (Applied Biosystems, Thermo Scientific, Waltham, MA, USA). Primer pairs were designed with PrimerBLAST (https://www.ncbi.nlm.nih.gov), and HPLC-purified primers were obtained from Eurofins (Eurofins Genomics, Luxembourg City, Luxembourg). The qPCR experimental workflow and data evaluation were carried out according to the MIQE (Minimum Information for Publication of Quantitative Real-Time PCR Experiments) guidelines [7] (see Text S3).

## Results and discussion

### Galactose excretion in lactose-grown precultures: initial observations

**Fig. 2:**
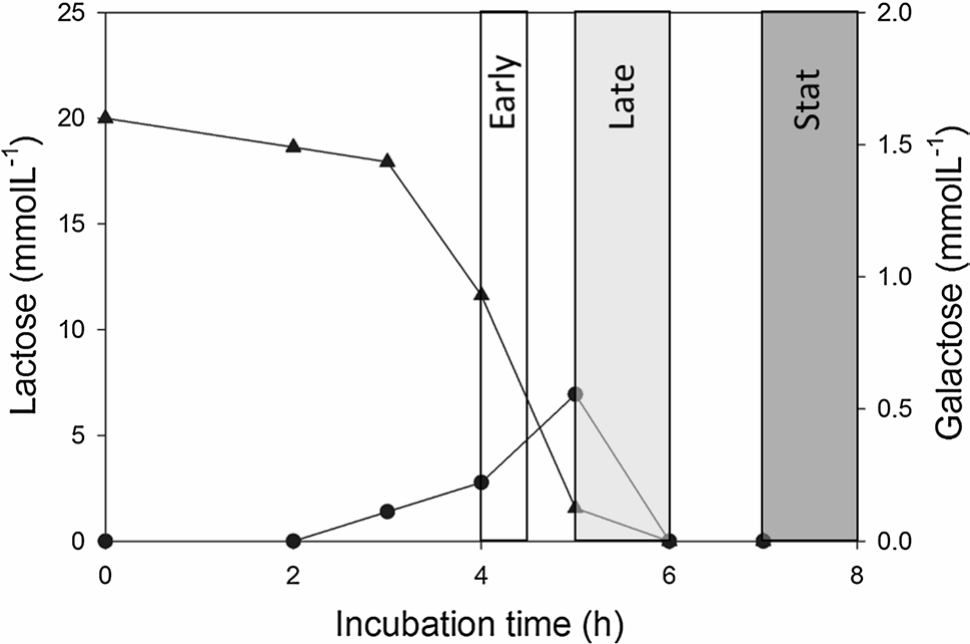
Galactose excretion in lactose-grown *L. lactis* anaerobic static flask precultures. Triangles: lactose concentration; circles: galactose concentrations. The times of preculture harvest are marked as Early—early exponential phase of the preculture, Late—late exponential phase of the preculture, Stat—stationary phase of the preculture

*Lactococcus lactis* cells grown in lactose-based medium in static flasks under anaerobic conditions were harvested at different incubation times for the inoculation of respiration-permissive bioreactor main cultures. Interestingly, the extracellular metabolite profiles from the lactose-grown precultures revealed the presence of galactose when lactose was almost fully consumed (Fig. 2, Table 1). Based on the growth status of the precultures, we divided the preculture samples into three distinct groups: (1) Early—early exponential phase of the preculture when galactose is not yet present in significant amounts in the preculture (4–4.5 h); (2) Late—late exponential phase of the preculture when galactose is being excreted while lactose is consumed (5.5–6 h); (3) Stat—stationary phase of the preculture when lactose is depleted and galactose is being consumed or has already been completely consumed (7–8 h).

**Table 1:** OD_600_, lactose concentration, galactose concentration, pH at the different times of preculturef harvest, and the length of the lag phases in subsequent main cultures

Preculture harvest time	Early—early exponential phase	Late—late exponential phase	Stat—stationary phase of the preculture
Incubation time (h)	4–4.5	5–6	7–8
OD_600_ (–)	0.8	1.23	2.27
pH (–)	6.6	6.4	5.0
Lactose (mmol L^−1^)	11.6	1.56	0
Galactose (mmol L^−1^)	0.22	0.55	0
Main culture lag phase (h)	3.5 ± 2	1 ± 0.5	7.5 ± 4

The times of preculture harvest are marked as Early—early exponential phase of the preculture, Late—late exponential phase of the preculture, Stat—stationary phase of the preculture. Subsequent main culture lag phases are given as the average ± standard deviation of at least five experiments

To investigate if the detected extracellular galactose could be due to hydrolysis of lactose to glucose and galactose via extracellular β-galactosidase, a pNP (*p*-nitrophenol) enzyme assay was used to determine the β-galactosidase activity in filtered cultivation medium. The intention was to obtain qualitative information on the contribution of possible extracellular enzyme activity. Samples from the different growth phases were assessed. None of the samples showed extracellular β-galactosidase activity (Table S1). We thus concluded that the extracellular galactose present in the medium was not the result of extracellular β-galactosidase activity but must instead have been excreted by the cells. These observations are in agreement with the generally known fact that *lactococci* used in dairy applications do not secrete significant amounts of lactose-degrading enzymes [25].

Additional NMR analysis of the main culture growth media confirmed the presence of extracellular galactose also under respiration-permissive and aerobic conditions (Table S2). After lactose depletion, galactose was rapidly metabolized in all conditions studied (Fig. S1). Galactose excretion during lactose consumption is well-known for *S. thermophilus* [19] and has previously been reported for the poorly respiring *L. lactis* subsp. *cremoris* (11, 19). Our results show that also the respiring strain *L. lactis* subsp. *lactis* CHCC2862 excretes galactose, and that the excreted galactose is metabolized after the lactose consumption, as is the case for *S. thermophilus* [20].

Galactose can be excreted to the extracellular medium either after hydrolysis of lactose by β-galactosidase in the Leloir pathway, or after dephosphorylation of galactose-6-phosphate formed in the tagatose pathway (Fig. 1). Although some evidence has been published for dephosphorylation under anaerobic conditions, galactose-6-phosphatase is not known in *L. lactis* [29]. A galactose–lactose antiporter system has been described in another LAB *S. thermophilus* [17, 20]. Based on sequence homology from *S. thermophilus*, it has been proposed that the LacS lactose permease in *L. lactis* IL1403 is a putative H^+^-lactose symporter or galactose–lactose antiporter [4]. *L. lactis* IL1403 has a comparable genome sequence to *L. lactis* subsp. *lactis* CHCC2862, the strain used in the present study [31]. A study on the lac-PTS-negative strain *L. lactis* IL1403 has also shown a functioning lactose permease and β-galactosidase system in lactose catabolism [1]. In lac-PTS-negative strains, lactose uptake depends on proteins encoded by genes of the lac operon: *lacS*, encoding a putative H^+^-lactose symporter or galactose–lactose antiporter; *lacA*, thiogalactoside acetyltransferase; and *lacZ*, β-galactosidase [4]. In the lac-PTS-negative *L. lactis* IL1403 strain the disruption of the *lacS* gene resulted in the loss of its lactose consumption ability [1].

### Aerobic and respiratory physiology on galactose

As previous studies have shown that anaerobic fermentation on galactose resulted in poor growth, with maximum specific growth rates (*µ*_max_) below 0.2 h^−1^ [9], controlled batch cultivations were performed using galactose under aerobic and respiration-permissive conditions to study *L. lactis* physiology in detail. In our study, aerobic and respiration-permissive conditions on galactose resulted in maximum specific growth rates of 0.63 h^−1^ and 0.7 h^−1^, respectively (Table 2). These maximum specific growth rates were significantly higher than the ones reported for galactose anaerobic conditions, but lower than the maximum specific growth rates on lactose under the same conditions (Table 2).

**Table 2:** Specific growth rates (*µ*_max_), yields (*Y*_i_) of products on consumed sugar (Cmol product/Cmol galactose or lactose), and carbon balances in bioreactor and flask batch cultures of *L. lactis* subsp. *lactis* CHCC2862 on galactose- or lactose-supplemented M17 medium, using aerobic or respiration-permissive conditions (average ± standard deviation of biological duplicates of bioreactor and flask experiments)

Culture conditions	*µ*_max_ (h^−1^)	*Y*_Lactate_	*Y*_Acetate_	*Y*_Acetoin_	*Y*_Biomass_	C-balance with biomass	C-balance without biomass
Galactose aerobic	0.63 ± 0.15	0.31 ± 0.02	0.42 ± 0.01	0.35 ± 0.00	0.33 ± 0.04	1.42	1.08
Galactose respiratory	0.70 ± 0.16	0.19 ± 0.0	0.42 ± 0.13	0.43 ± 0.01	0.37 ± 0.07	1.42	1.05
Lactose aerobic	0.95 ± 0.03	0.77 ± 0.01	0.06 ± 0.01	0.11 ± 0.01	0.18 ± 0.00	1.12	0.94
Lactose respiratory	0.86 ± 0.05	0.36 ± 0.02	0.06 ± 0.01	0.60 ± 0.02	0.2 5 ± 0.01	1.27	1.02

**Fig. 3:**
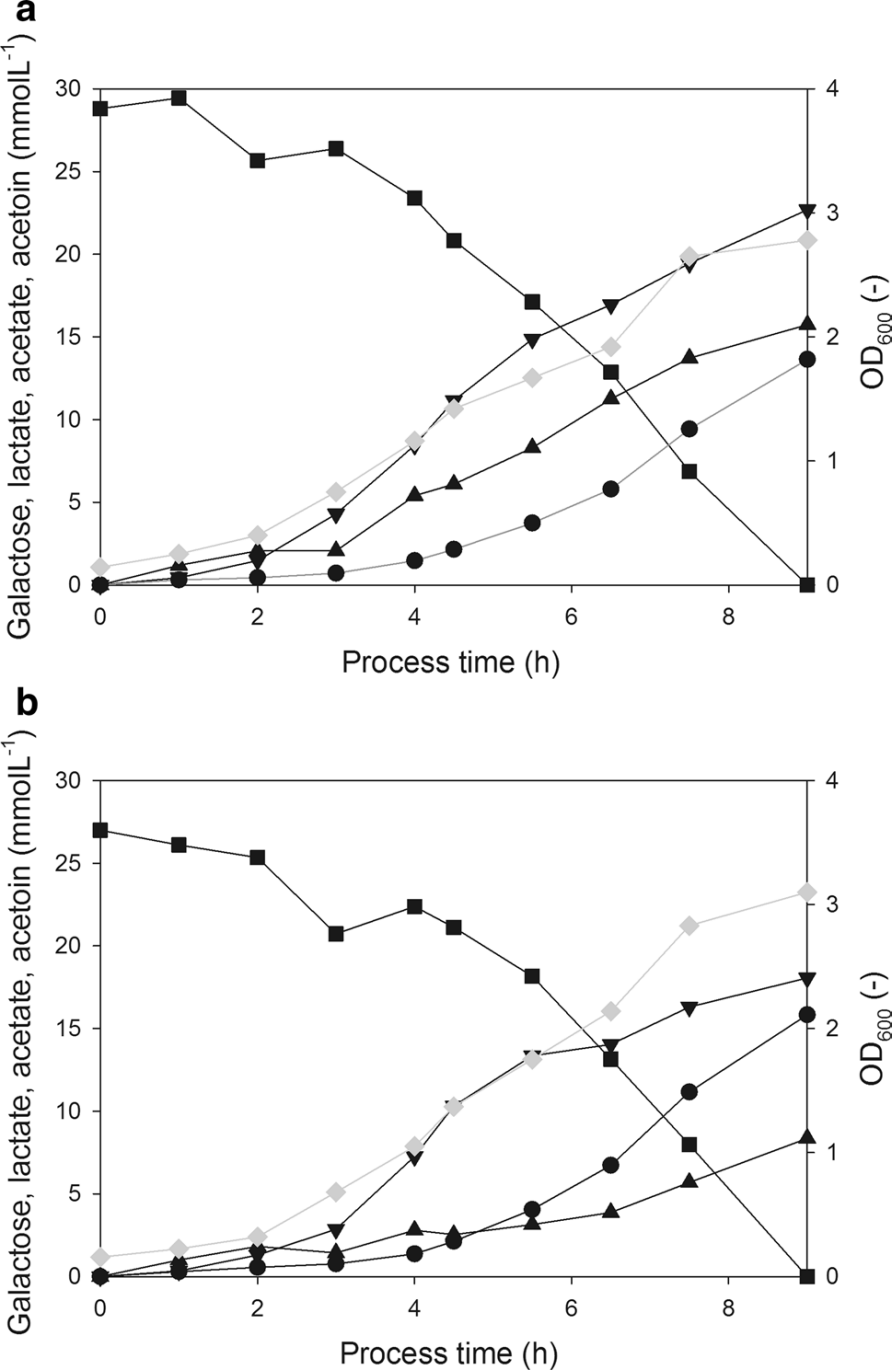
OD_600_ and extracellular metabolite concentrations in aerobic (**a**) and respiration-permissive hemin-supplemented (**b**) batch cultures of *L. lactis* subsp. *lactis* CHCC2862 on galactose. Black squares: galactose; black triangles: lactate; black upside down triangles: acetate; black circles: acetoin; grey diamonds: OD_600_. Representative values of two biological replicates

When cultured on galactose, *L. lactis* undergoes heterofermentation and produces acetate, formate, and ethanol as end products, in addition to lactate [36]. Respiration-permissive galactose-grown cultures showed higher biomass yields while lactate production was lower compared to aerobic galactose-grown cultures (Fig. 3a, b). The lactate production was about 50% lower during respiration-permissive growth than during aerobic growth without hemin supplementation. These changes in the biomass yield and the extracellular metabolite pattern are consistent with previous observations reported for lactose-grown cultures under aerobic conditions, with or without hemin supplementation [31].

Catabolism of lactose and glucose in LAB is a tightly regulated mechanism, in which the catabolite control protein (CcpA) is the central regulator of glucose and lactose assimilation by controlling the expression of the lac-PTS and the tagatose pathway genes [28]. The lac-PTS and the concomitant ATP-dependent phosphorylation is a prerequisite for the function of CcpA [11, 13]. The metabolism of galactose is repressed by CcpA in the presence of lactose and glucose [28]. It has been reported that CcpA regulation is also important in respiratory metabolism, as CcpA controls the shift from fermentation to respiration by regulating the hemin uptake [14]. Respiratory metabolism has previously been confirmed using both glucose and lactose. Our results indicate that during galactose utilization, the cellular metabolism can also shift towards respiration with hemin addition.

Surprisingly, the biomass yield on consumed galactose was higher than that on lactose, under both aerobic and respiration-permissive conditions (Table 2). Previous publications have demonstrated a twofold higher biomass concentration in respiration-permissive glucose-grown cultures on M17 complex medium than in anaerobic cultures [15, 24, 31]. However, none of these studies provided quantitative assessments of the sugar consumption. Being a fastidious microorganism, *L. lactis* uses amino acid building blocks for biomass formation, while sugar catabolism provides ATP for biosynthesis [30]. This means that the contribution of amino acids to the biomass formation is also significant when calculating biomass yields on sugar consumption.

The higher biomass yield on galactose than on lactose (Table 2) led us to speculate that growth on galactose is energetically beneficial under aerobic and respiration-permissive conditions as: (1) the higher acetate production on galactose results in a higher yield of ATP on consumed sugars [9, 30], and (2) galactose metabolism plays a positive role in aerobic growth and increase aerotolerance, leading to higher biomass yields [14, 34].

**Fig. 4:**
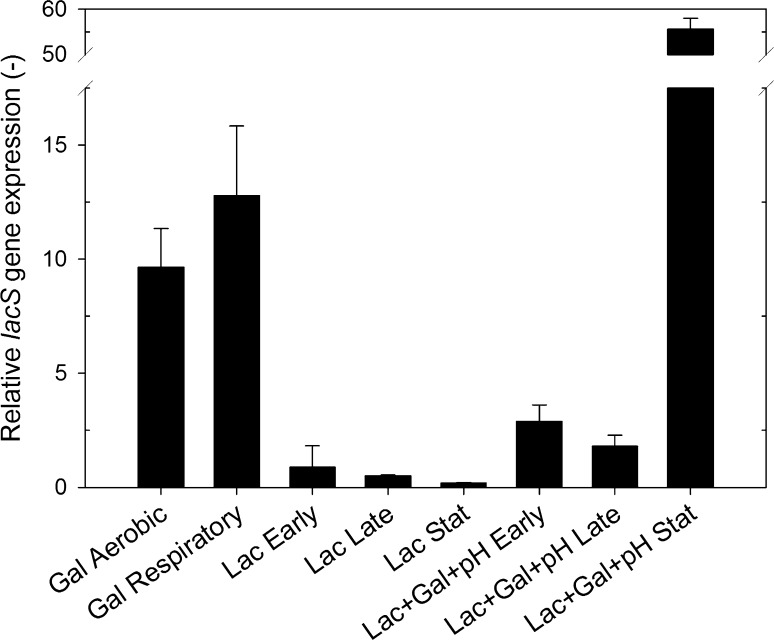
Relative *lacS* gene expressions using *rluD* as the reference gene assuming 100% primer efficiency (Text S3). Samples from aerobic (Gal Aerobic) and respiration-permissive galactose–grown (Gal Respiratory) cultures, lactose-grown anaerobic precultures (Lac), and pH-controlled preculture on lactose and initially supplied galactose (Lac + Gal + pH), at Early, Late, and Stat harvest points

In addition to being a putative galactose–lactose antiporter and H^+^-lactose symporter [3], sequence analysis of *L. lactis* subsp. *cremoris* has shown 96% identity between its galactose permease (*galA*) and the *lacS* of *L. lactis* IL1403, indicating the potential galactose permease function of LacS [16]. Furthermore, LacS in *L. lactis* IL1403 has been shown to have a higher affinity for galactose than for lactose [1]. Therefore, the putative H^+^-lactose symport activity of LacS plays only a minor role in lactose transport in *L. lactis* IL1403 [1]. To gain some information on the expression of *lacS* as a response to different carbon sources, gene expression analysis using qPCR was performed. In the absence of lactose, the *lacS* gene was strongly upregulated in samples from galactose-grown batches, and *lacS* expression was significantly higher under respiration-permissive than under aerobic conditions (Fig. 4, Text S3). The expression of *lacS* was ten-fold higher in galactose-grown cultures than in cells harvested from precultures on lactose (Fig. 4). The strong correlation between *lacS* gene expression and galactose consumption in the cultures indicates that LacS has a role in galactose uptake.

### Relation between the length of the lag phase and the presence of galactose excretion

The harvest times of the precultures were divided into the “Early”, “Late” and “Stat” groups based on the microbial growth status (Fig. 2). Interestingly, the duration of the lag phase of the main cultures varied depending on the time of harvest of the preculture. Surprisingly, the “Late” precultures, which were harvested after 5.5–6 h when both lactose and galactose were present, showed short and consistent lag phase when used to inoculate the subsequent main cultures. When precultures were harvested earlier (“Early”) or later (“Stat”) than the “Late” preculture, the lag phases in the main cultures were longer and more inconsistent and varied between 3.5 ± 2 h and 7.5 ± 4 h (Table 1).

**Fig. 5:**
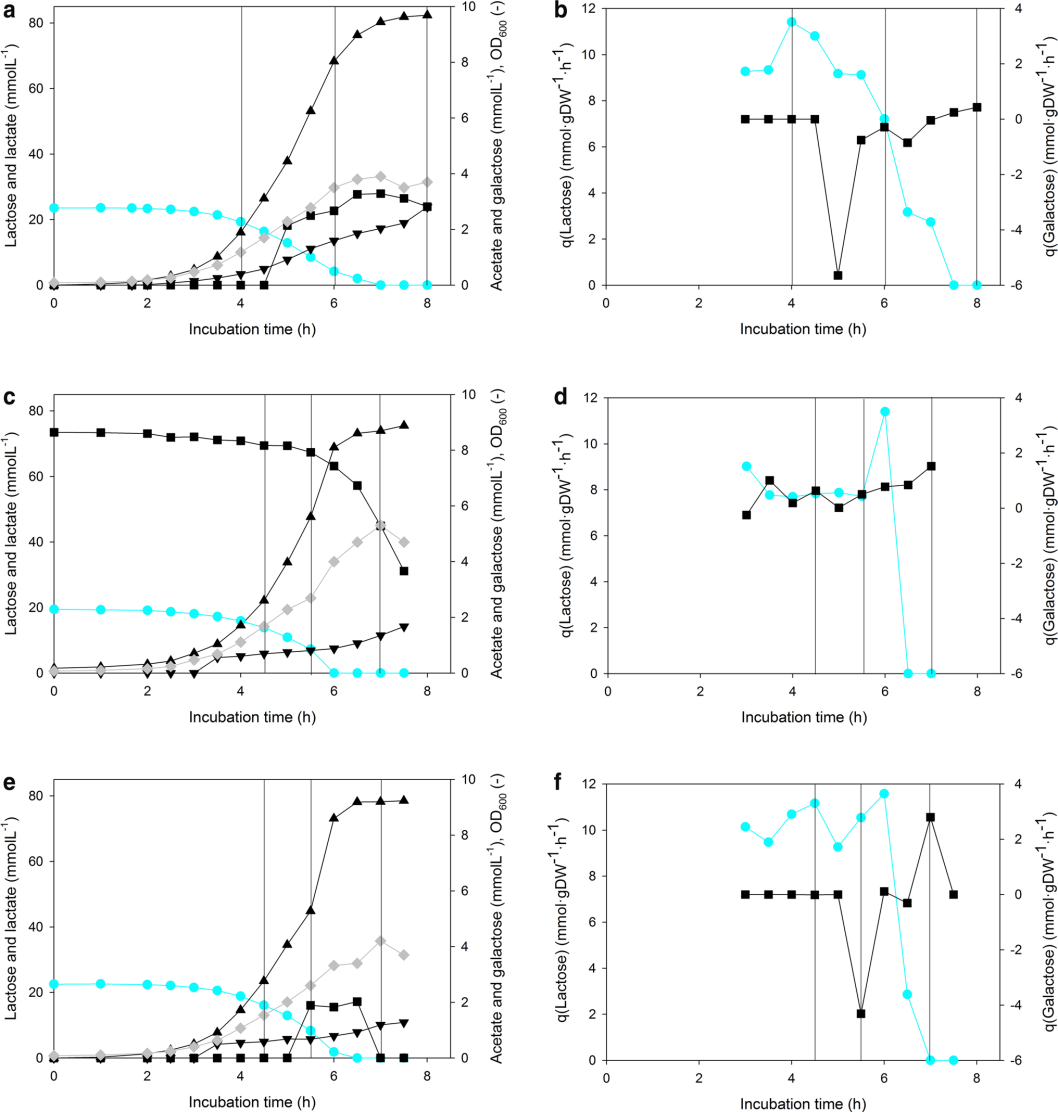
Concentration profiles (**a**, **c**, **e**) and specific sugar consumption rates (**b**, **d**, **f**) in lactose-grown anaerobic precultures (Lac) without pH control (**a**, **b**), pH-controlled precultures on lactose and initially supplied galactose (Lac + Gal + pH) (**c**, **d**), and pH-controlled precultures on lactose (Lac + pH) (**e**, **f**). The vertical black lines indicate the time of inoculum harvest for main cultures. **a**, **c**, **e** Blue circles: lactose; black triangles: lactate; grey diamonds: OD_600_; black upside down triangles: acetate; black squares: extracellular galactose. **b**, **d**, **f** Blue lines and circles: Specific lactose consumption rates (q(Lactose), mmol g_DW_^−1^ h^−1^); black lines and squares: specific galactose consumption rates (q(Galactose), mmol g_DW_^−1^ h^−1^). Representative values of two biological replicates

The results led us to formulate the hypothesis that the length of the lag phase of the main culture is related to the presence of galactose in the preculture. To investigate this hypothesis, we used high-performance bioreactors for both precultures and main cultures, and precultures were again harvested at the Early, Late, and Stat time points. Precultures were grown anaerobically either on lactose with pH monitoring but without pH adjustment (called “Lac”) to simulate previously ran anaerobic shake flask precultures, or pH controlled at pH 6.0 with galactose initially added to the medium in addition to lactose (“Lac + Gal + pH”). The extracellular metabolites, i.e., high lactic acid production, no acetoin production confirmed the anaerobic nature of the precultures in bioreactors (Fig. 5). Although the experiments were performed in laboratory scale, this two-step cultivation strategy mimics the industrial scale propagation and production of dairy starter cultures. The subsequent lag phase lengths of the preculture conditions are given in Table 3. Inocula from “Lac” precultures had different behavior depending on the harvest time. “Early” and “Stat” inocula resulted in lag phases of 2.4–4.5 h, whereas “Late” inocula gave reproducible and consistently short 1 h lag phases. When galactose was initially supplemented to the preculture (Lac + Gal + pH), the lag phase was always below 1 h, regardless of the time of harvest of the preculture.

**Table 3:** The length of the lag phase of the main cultivations using inoculum from the different anaerobic precultures at different harvest times (Early—early exponential phase, Late—late exponential phase, Stat—early stationary phase)

Preculture harvest time	Lactose-grown (Lac)		Lactose-grown with galactose addition pH control (Lac + Gal + pH)	Lactose-grown with pH control (Lac + pH)
	Inoculum pH	Lag phase (h)	Lag phase (h)	Lag phase (h)
Early (4–4.5 h)	6.24–6.45	4.5 ± 3.4	1 ± 0.0	5.5 ± 4.2
Late (5.5–6 h)	4.97	1.0 ± 0.4	1 ± 0.0	9.8 ± 0.8
Stat (7–8 h)	4.72–4.77	2.4 ± 2.7	0.8 ± 0.4	6.5 ± 3.5

The length of the lag phase was determined based on the first derivative of the CO_2_ (%) signal (Text S1). Main culture lag phases are given as the average ± standard deviation of at least three separate pairs of precultures and main cultures

The growth of the two preculture types was very similar, each reaching OD ≈ 4 at the time of lactose depletion (Fig. 5a, c). Yields and carbon balances of the precultures are found in Table S3. Galactose was excreted in the “Lac” precultures and reached a level of 3.3 mmol L^−1^. When the pH was controlled in the “Lac + Gal + pH” preculture galactose was consumed after lactose depletion, whereas in the uncontrolled “Lac” preculture the galactose remained in the medium. In the “Lac” precultures, the start of galactose excretion and the decrease in the specific lactose uptake rate coincided (Fig. 5b, after 5.5 h incubation). Although lactose was still available, the specific lactose uptake rate in “Lac” decreased, probably due to the decrease in pH (Table 1) [27]. This decrease in specific lactose uptake rate towards the end of the lactose consumption phase (Fig. 5b) suggests a limitation in either the lactose uptake or in lactose metabolism. Lac-PTS is known to exhibit high affinity to lactose [39], and is therefore not a plausible explanation to the decrease in the specific lactose uptake rate. Considering limitations in the lactose metabolism, one possible reason for the decrease in the specific lactose uptake can be that lactose assimilation flux via the lac-PTS may suffer from shortage of PEP through the glycolytic flux. When the extracellular lactose concentration reached 13 mM, galactose excretion started, leading to a final concentration of 3.3 mM (Fig. 3a). Assuming equimolarity of the galactose–lactose antiporter mechanism, excreting 3.3 mM galactose corresponds to the uptake of 3.3 mM lactose. This could suggest that in that late phase of cultivation, when galactose is excreted, lactose permease is responsible for the uptake of 25% of the residual lactose, while the rest is still internalized via the PEP-dependent lac-PTS transporter in lac-PTS-positive *L. lactis* [33]. In Lac + Gal + pH preculture, the specific lactose uptake rate was maintained until lactose depletion, after which the galactose was consumed (Fig. 5d).

The expression levels of the gene *lacS* were determined by qPCR, using *rluD* (Fig. 4) and *rpoD* (data not shown) as reference genes, at the Early, Late, and Stat time points of the precultures. The pattern of expression levels of *lacS* were different in the two preculture conditions (Fig. 4). In Lac precultures *lacS* was expressed at Early and Late sampling points (Fig. 4), with a concomitant decrease in specific lactose uptake rate (Fig. 5b) and accumulation of extracellular galactose (Fig. 5a). Under Stat conditions in the Lac preculture five-fold lower relative expression of *lacS* was observed compared to the expression at the Late sampling point. At the Stat sampling point, after the lactose depletion, the low pH supposedly limited the rate of galactose uptake, which correlates with the low *lacS* expression.

In Lac + Gal + pH precultures galactose was supplemented and the relative gene expression of *lacS* was higher than in the Lac preculture in all of the samples (Fig. 4). In Lac + Gal + pH preculture, lactose was being consumed in the presence of added galactose in both the Early and the Late samples. In the Stat sample of Lac + Gal + pH preculture *lacS* was highly expressed. In this sample lactose had been consumed, but the supplemented galactose was still present and being consumed. The data, therefore, indicate that the presence of extracellular galactose relates to enhanced *lacS* expression. Similar high increase of *lacS* expressions were observed during solely galactose-grown cultures (Fig. 4). The gene expression data and the analysis of metabolic pathways and specific sugar uptake rates suggested that the galactose–lactose antiporter mechanism was activated and *lacS* also functioned as a galactose permease.

In previous studies on the cultivation of *L. lactis* under respiration-permissive conditions, precultures were either grown until the stationary phase [31] or, more commonly, using fresh dilutions of overnight stationary cultures [34, 38]. In these, the preculture pH was neither monitored nor controlled. In bioreactor studies of *L. lactis* main cultivations, depending on the application and the cultivation conditions, the culture pH values were controlled in the range of 5.8–6.6 [5, 23, 32, 40]. In our study, respiration-permissive main cultivations and Lac + Gal + pH-controlled anaerobic precultures were carried out at pH 6.0. However, when the preculture pH was not controlled, the pH of the Lac preculture samples varied with the harvest time (Table 1, Table 3). To rule out the effect of preculture pH on the lag phase of the main culture, pH-controlled anaerobic preculture experiments were carried out using lactose (Lac + pH preculture, Fig. 5e, f). All three harvest points in Lac + pH preculture showed long lag phases in the main cultivations (5.5–9.8 h, Table 3). In Lac + pH preculture, the time resolution of sampling (30 min) was in the same range as the time frame in which the extracellular galactose was present and was rapidly metabolized. Moreover, as Lac + pH preculture was pH-controlled, the volumetric lactose uptake did not decrease due to the acidification (Fig. 5f). Less galactose was excreted in Lac + pH preculture than in Lac preculture where the pH was not controlled (Fig. 5a, e). This indicates that slower lactose uptake caused by lower pH, i.e., acidity, and the presence of lactose activates LacS, which has been described as a putative galactose–lactose antiporter or H^+^-lactose symporter. If the pH of the culture is not controlled and it becomes more acidic, the cells will have to expend more ATP (via H^+^-ATPase) in maintaining the intracellular pH [12], or to use proton symporters for sugar uptake. The permease LacS may be favored over lac-PTS for lactose uptake as the lactose assimilation flux via the lac-PTS may suffer from shortage of PEP due to a decrease in the specific lactose uptake rate, and hence the glycolytic flux, towards the end of the lactose consumption phase. LAB strains that use LacS to compete for the available lactose and to enable rapid growth might have an evolutionary advantage in acidic environments. On the other hand, at low pH galactose was not consumed (Lac preculture) indicating pH inhibition of the galactose permease function, whereas in both in Lac + pH and Lac + Gal + pH precultures, where pH was controlled, galactose was rapidly assimilated after lactose depletion.

Versatility in the sugar transport systems can lead to a shortened lag phase and thus can be a competitive advantage for an opportunistic microorganism. It has recently been shown, by model simulations, that an optimal distribution between permease- and PTS-mediated glucose uptake leads to the highest glucose uptake rates in obligate heterofermentative LAB [22]. Having the flexibility to use two sugar uptake systems may be especially beneficial during adaptive phases, where various ATP-requiring signaling pathways must be allowed to take place, and rates of metabolic pathways and intracellular concentrations of metabolites may fluctuate. Flexible sugar uptake, that can be maintained despite variations in intracellular PEP and ATP concentrations and pH, may be a key to robust and competitive microbial growth. Our gene expression results suggested that the lactose permease (*lacS*) is induced by a decrease in the lactose uptake rate and the presence of external galactose in lactose-grown lac-PTS positive *Lactococcus lactis* subsp. *lactis* cultures. Main cultures inoculated by precultures harvested under these cultivation conditions had short and reproducible lag phases. Therefore, extracellular galactose, indicating the presence of the *lacS* permease in parallel with the lac-PTS, is a potential biomarker for harvesting cells for optimal starter culture performance.

## Conclusions

The results of controlled respiration-permissive batch cultivations of *L. lactis* in the present study showed that it is important to harvest the preculture at a specific time, i.e., at a certain physiological state in the late exponential phase, to minimize the lag phase of the main culture. We found that galactose in lactose-grown cultures was excreted and consumed after the lactose depletion as long as no inhibition by the low pH was present. Galactose, excreted or externally added is shown to be essential to produce a *Lactococcus lactis* subsp. *lactis* inoculum that results in a short and reproducible lag phase of the main cultivation (≤ 1 h lag phase). The presence of galactose in the precultures of fast lactose fermenting starter culture dairy strains provides a measurable physiological marker for short culture lag phase in subsequent lactose-grown cultures. Analyzing the specific sugar uptake rates, we found that the start of galactose excretion and the decrease in the specific lactose uptake rate coincided. Furthermore, gene homology and the upregulation of *lacS* suggested that LacS is responsible for the excretion of galactose in *L. lactis* subsp. *lactis* as a galactose–lactose antiporter. Also, in the absence of lactose, in galactose-grown cultures higher *lacS* expression pointed to an additional galactose permease function of LacS. Studying the factors affecting lactose and galactose utilization in *L. lactis* can contribute to engineering strategies to create more efficient industrial strains.

## Electronic supplementary material

Below is the link to the electronic supplementary material.

Supplementary material 1 (PDF 749 kb)
