# Supplementary material for: Presence of galactose in precultures induces lacS and leads to short lag phase in lactose-grown Lactococcus lactis cultures
Source: J Ind Microbiol Biotechnol. 2018 Nov 9;46(1):33–43. doi: 10.1007/s10295-018-2099-0 (PMC6339885; doi:10.1007/s10295-018-2099-0)
Supplement: Supplementary file 1 — Supplementary material 1 (PDF 749 kb) [file 10295_2018_2099_MOESM1_ESM.pdf]

Supplementary material for

Title

Presence of galactose in precultures induces *lacS* and leads to short lag phase in lactose-grown *Lactococcus lactis* cultures

List of Authors

Bettina Lorántfy[1], Anna Johanson[1,2], Fábio Faria-Oliveira[1], Carl Johan Franzén[1], Valeria Mapelli[1], Lisbeth Olsson[1,*]

[1]Division of Industrial Biotechnology, Department of Biology and Biological Engineering, Chalmers University of Technology, SE-412 96 Gothenburg, Sweden

[2]Chr. Hansen A/S, DK-2970 Hørsholm, Denmark

[*]Corresponding author: e-mail: lisbeth.olsson@chalmers.se, phone: +46317723805

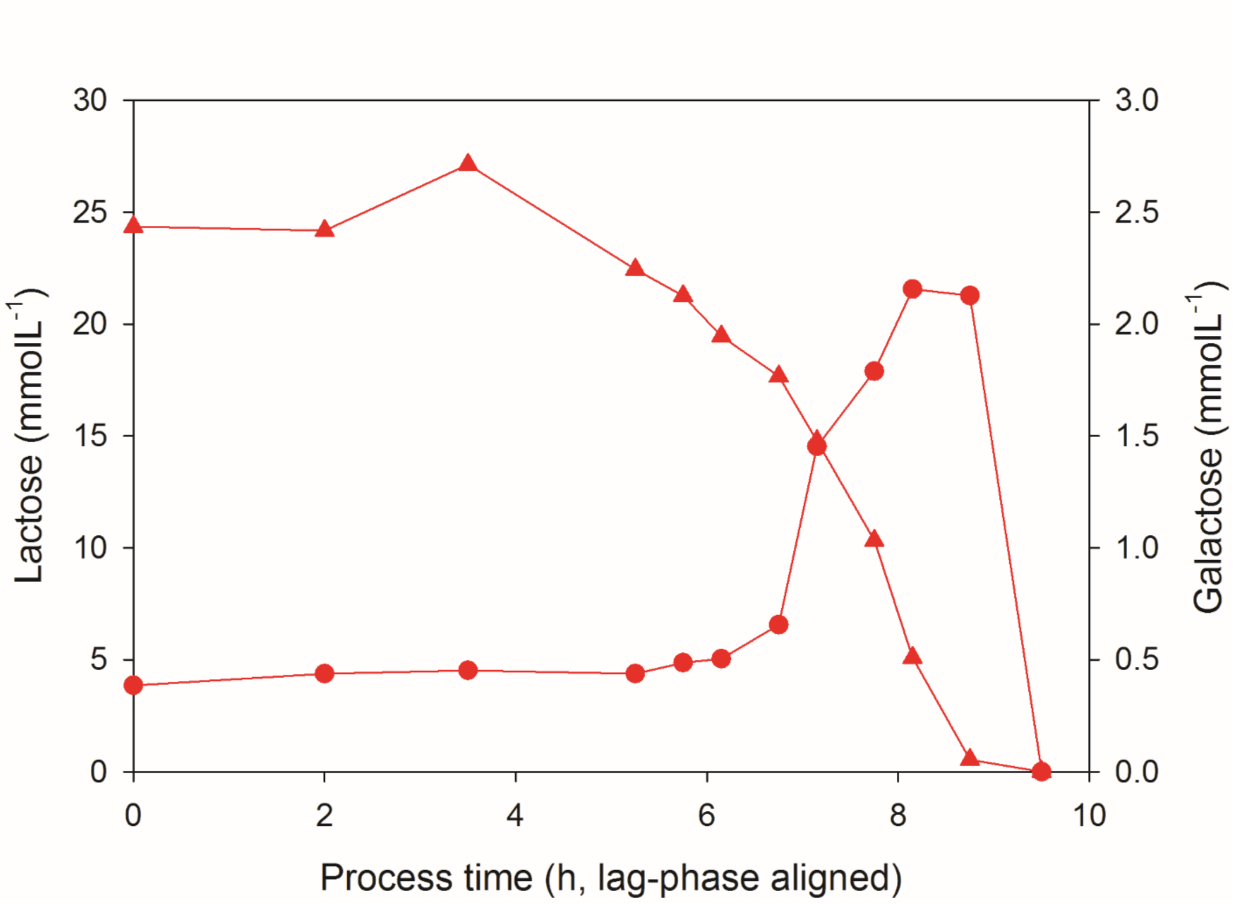

Supplementary Figure S1. Galactose excretion and re-consumption in lactose-grown respiration-permissive (aerobic hemin-supplemented) *L. lactis* bioreactor main cultures. Triangles: lactose concentration; Circles: galactose concentration. Culture lag phase is not shown.

Supplementary Table S1 β-galactosidase assay results. Bioreactor batch samples were assessed along with the necessary positive and negative controls to rule out the contribution of extracellular β-galactosidases in galactose excretion (mean values of two technical replicates from two biological replicates, respectively). The relatively higher absorbance values in bioreactor samples from the later phase of growth can be interpreted as the increasing levels of compounds interfering with the assay towards the end of the lactose phase, such as lactate, ethanol, and acetate.

**Sample and description**	**Absorbance at 410 nm**
Fresh M17 medium (negative control)	0.220
w/o pNP bioreactor sample 1	0.195
Bioreactor sample 1 – Early - early exponential phase (galactose not yet excreted)	0.215
w/o pNP bioreactor sample 2	0.226
Bioreactor sample 2 – Late - late exponential phase (galactose excretion)	0.234
Bioreactor sample 3 – Stat - stationary phase	0.283
100x diluted β-galactosidase (positive control)	1.527

Supplementary Table S2 Culture media galactose analysis by ^{1}H-NMR Spedia-NMR^{TM}. The analytics was purchased from Spinnovation Analytical integrated NMR services, Oss, the Netherlands.

condition	sample description	galactose (mM)
**Respiration-permissive (aerobic hemin-supplemented)**	inoculation	<LOD
	Early - early exponential phase	<LOD
	Late - late exponential phase	0.75
		0.80
		1.09
	Stat – stationary phase, at lactose depletion	1.60
**aerobic**	inoculation	<LOD
	Late - late exponential phase	0.80
		1.32
		1.61
	Stat – stationary phase, at lactose depletion	1.89

Supplementary Table S3 Quantitative summary of the anaerobic preculture bioreactor experiments. Cmol/Cmol yields and carbon balances of the lactose growth phase for anaerobic lactose grown precultures (mean values of biological duplicates with standard deviations).

Propagation culture	Lactate	Acetate	Ethanol	Biomass	C-balance with biomass	C-balance without biomass
**"Lac" Lactose -grown**	0.88±0.02	0.02±0.01	0.01±0.01	0.18±0.02	1.09	0.91
**"Gal+Lac" Lactose grown with pH control and galactose addition**	0.88±0.02	0.02±0.01	0.00±0.01	0.23±0.01	1.13	0.90
**"Lac pH" Lactose-grown with pH control**	0.88±0.02	0.02±0.01	0.01±0.01	0.17±0.01	1.08	0.91

Supplementary Text S1 Off-line analytics of bioreactor cultures.

Off-line analyses

Biomass concentration

The optical density (OD_{600}) was measured in polystyrene semi-micro cuvettes using a Genesis 20 spectrophotometer (Thermo Scientific, Waltham, MA, USA) in its linear range. The conversion factor between dry weight (DW) and optical density were determined using cell samples from cells grown in shake flasks. 5 mL of the broth volume was harvested at the early stationary phase of growth, vacuum filtered through a filter disc (0.45 µm PESU Membrane, Sartorius Stedim Biotech GmbH, Göttingen, Germany) and subsequently washed with 2x5 mL deionized water. Dry weight measurements were performed in both biological and technical triplicates. The conversion factor was found to be 0.38±0.03 g DW L^{-1} $(OD_{600})^{-1}$.

Extracellular metabolites

Lactose, galactose and extracellular metabolites were analyzed using HPLC (Dionex Ultimate 3000 unit, Thermo cientific, Waltham, MA, USA) with an Aminex HPX-87P 300 x 7.8 mm column (Bio-Rad, Hercules, CA, USA). The oven temperature was set to 80°C and the eluent was 5 mM H_2SO_4 at a flow rate of 0.6 mL min^{-1}. A Shodex RI-101 refractive index detector (Showa Denko K.K., Tokyo, Japan) was used to quantify the sugars and the extracellular metabolites. Galactose was also analyzed using NMR (Spedia-NMR™, Spinnovation Analytical integrated NMR services, Oss, the Netherlands) to confirm the HPLC results (Table S2).

Calculation of yields, rates and carbon balances

Based on the HPLC data of the consumed lactose and the produced extracellular metabolites, the extracellular metabolite yields were calculated as Cmol/Cmol yields. The measured amount of extracellular

metabolite produced at the time of lactose depletion was divided by the measured initial lactose concentration. For the biomass yield, the measured g L^{-1} DW biomass concentration at the time of lactose depletion was converted to Cmol using previously published biomass composition and molar mass of *L. lactis* [2]. The same biomass composition was used when lactose and galactose were applied, and under anaerobic, aerobic and respiration-permissive conditions. Specific sugar consumption rates of lactose and galactose were calculated using the volumetric sugar consumption rates and the biomass dry weight concentrations (Fig. 5B, D, F). Carbon balances on lactose consumed were calculated as the sum of the Cmol/Cmol yields of the extracellular metabolites produced (lactate, acetate, acetoin).

Calculation of the off-gas rates

The volumetric carbon dioxide evolution rate (CER, mmol (L h)$^{-1}$) and oxygen uptake rate (OUR, mmol (L h)$^{-1}$) were calculated as

$$OUR\ (mmol(Lh)^{-1}) = \frac{F\ (Lh^{-1})}{22.41\ (Lmol^{-1})} \cdot \left[0.2095 - \left(r_{inert} \cdot \frac{oxygen\ (\%)}{100\ (\%)}\right)\right] \cdot \frac{1000}{V\ (L)} \tag{1}$$

$$CER\ (mmol(Lh)^{-1}) = \frac{F\ (Lh^{-1})}{22.41\ (Lmol^{-1})} \cdot \left[\left(r_{inert} \cdot \frac{carbon\ dioxide(\%)}{100\ (\%)}\right) - 0.0004\right] \cdot \frac{1000}{V\ (L)} \tag{2}$$

$$x_{H_2O} = \left(0.2095 - \frac{oxygen\ wet(\%)}{100(\%)}\right)/0.2095 \tag{3}$$

$$r_{inert} = (1 - 0.2095 - 0.0004)/\left(1 - \frac{oxygen\ (\%)}{100\ (\%)} - \frac{carbon\ dioxide(\%)}{100\ (\%)} - x_{H_2O}\right) \tag{4}$$

where *F* (L h^{-1}) is the volumetric aeration rate at 1 atm and 20 °C, 22.41 (L mol^{-1}) is the molar gas volume at 1 atm and 20 °C, 0.2095 is the oxygen molar fraction in the inlet air, r_{inert} is a compensation factor to relate the outlet gas flow rate to the aeration rate based on an inert gas balance, 0.0004 is the carbon dioxide molar fraction in air, and *V* (L) is the reactor volume. In order to compensate for the moisture content of the off-gas, the moisture fraction of the off-gas (x_{H_2O}) was approximated using the *oxygen wet*

(%) value. The *oxygen wet* (%) was the measured oxygen content of the off-gas before inoculation in batch cultures.

Supplementary Text S2 Lag phase analysis of the main bioreactor cultures.

Lag phase calculations

The end of the lag phase was determined as the time when the first derivative of the CO_2 (%) signal average had a non-zero value. The CO_2 (%) and O_2 (%) in the off-gas were monitored continuously using BlueSens gas analyzers (BlueSens Gas Technology GmbH, Herten, Germany) to calculate the volumetric CO_2 evolution rate (CER, $mmol \cdot L^{-1} \cdot h^{-1}$) and the O_2 uptake rate (OUR, $mmol \cdot L^{-1} \cdot h^{-1}$).

Table S2.I

Main culture lag phases and preculture harvest times (depending on the microbial status of the preculture, Early – early exponential phase (4-4.5 h) ii) Late – late exponential phase (5.5-6 h) iii) Stat – stationary phase (7-8 h)) for the three different anaerobic precultures in bioreactors: Lac) without pH control, monitoring only, Lac+Gal) pH control at pH 6.0 and with an additional 0.15% (w/v) galactose, Lac pH) pH control at pH 6.0. The main cultures are marked as A-P when inoculated by Lac preculutres, Q-W when inoculated by Gal+Lac and X-Ö when inoculated by Lac pH precultures.

**Lac**				**Gal+Lac**				**Lac pH**			
**Preculture harvest time (h)/main cultivation lag phase (h)**											
**Early**	4-4.5 h	A	4	**Early**	4.5 h	Q	1	**Early**	4.5 h	X	8.5
		B	1			R	1			Y	2.5
		C	7	**Late**	5.5 h	S	1	**Late**	5.5 h	Z	9.6
		D	1			T	1			Å	10
		E	7	**Stat**	7.0 h	U	0.5	**Stat**	7.5 h	Ä	9
		F	9.6			W	1			Ö	4
		G	0.5								

		H	5.6
**Late**	6 h	C	0.5
		D	1.5
		I	1
		J	1
		K	1
**Stat**	7.5-8 h	E	1
		F	8
		L	1.5
		M	2.6
		N	0.6
		O	1
		P	2

Examples of main cultures

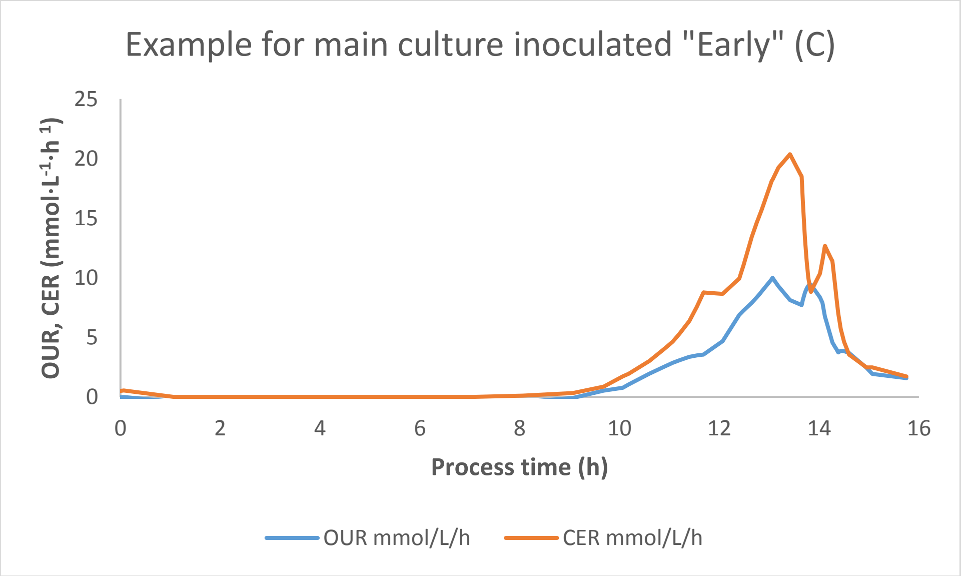

Figure S2.1. Example for main culture inoculated Early by Lac precultures. Preculture harvest times depending on the microbial status of the preculture were categorized as Early – early exponential phase (4-4.5 h) ii) Late – late exponential phase (5.5-6 h) iii) Stat – stationary phase (7-8 h).

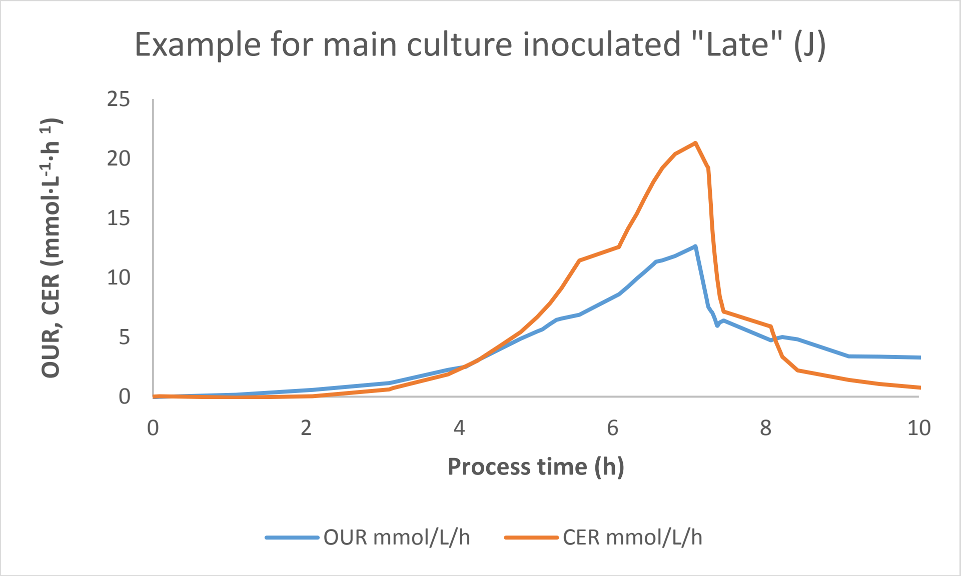

Figure S2.2. Example for main culture inoculated Late by Lac precultures. Preculture harvest times depending on the microbial status of the preculture were categorized as Early – early exponential phase (4-4.5 h) ii) Late – late exponential phase (5.5-6 h) iii) Stat – stationary phase (7-8 h).

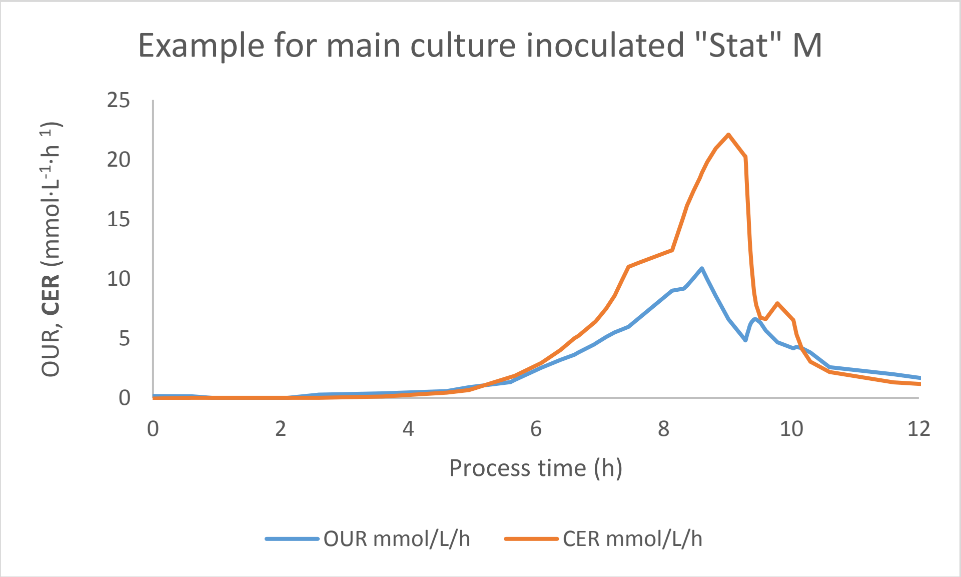

Figure S2.3. Example for main culture inoculated Stat by Lac precultures. Preculture harvest times depending on the microbial status of the preculture were categorized as Early – early exponential phase (4-4.5 h) ii) Late – late exponential phase (5.5-6 h) iii) Stat – stationary phase (7-8 h).

Supplementary Text S3 MIQE (Minimum Information for Publication of Quantitative Real-Time PCR Experiments).

A) Experimental design

Goal of experiment:

- *lacS* gene expression analysis in galactose-grown aerobic and respiration-permissive batch *L. lactis* cultures
- In the anaerobic *L. lactis* bioreactor preculture samples: to compare the expressions of the *lacS* over time; Early, Late, Stat sampling.

B) Sampling

Sampling for RNA extractions were performed in technical duplicates from biological duplicate batch bioreactor cultures. Samples from batch cultures supplemented with galactose were collected at the time of the respiratory switch, when hemin intake for respiration becomes relevant [1](at 5.5 h, Fig. 3B). The aerobic samples from batch cultures supplemented with galactose were collected at the same time as the samples from the respiration-permissive (aerobic hemin-supplemented) samples (Fig. 3A) in order to ensure a similar range of biomass concentration. Samples from the anaerobic bioreactor precultures were harvested at different time points (indicated in the Results section) in different volumes in order to ensure a similar range of total biomass for the RNA extraction step. With a suitable OD_{600} value, 0.5-1 mL culture volume was immediately vortexed for 5 s with 1 mL RNAprotect Bacteria Reagent (Qiagen N.V., Venlo, the Netherlands). Afterwards, the mixture was incubated for 5 min at room temperature, and then centrifuged for 10 min 5000 g (Allegra 25R centrifuge, Beckman Coulter, Brea, CA, USA). The supernatants were decanted and residual supernatant was removed by gently dabbing the inverted tube once onto a paper towel. The sample pellets were snap-frozen by liquid N_2 for further -80°C storage.

C) RNA extraction

For the RNA extractions, the Qiagen RNeasy Mini kit spin columns were applied with Qiagen RNase Free DNase Set for on-column DNA digestion (Qiagen N.V., Venlo, the Netherlands). Due to the high DNA load in the RNA samples, the Qiagen protocols of "Enzymatic lysis and proteinase K digestion of Bacteria", "Purification of total RNA from Bacterial Lysate", "Optional On-Column DNase Digestion Using the RNase Free DNase Set" had to be slightly modified to the experimental conditions.

RNA extraction protocols Fusion of Qiagen protocols of:

- RNAprotect Bacteria Reagent Protocol 4: **Enzymatic lysis and proteinase K digestion of Bacteria**
- RNeasy Mini kit Protocol 7: **Purification of total RNA from Bacterial Lysate**
- RNase free DNase Set: **Optional On-Column DNase Digestion Using the RNase Free DNase Set**

The samples were treated with RNAprotect reagent and frozen at -80°C.

Necessary chemicals: EtOH absolute, 2-Mercaptoethanol, Qiagen RNeasy Mini Kit, Qiagen DNase Set, TE buffer (TE buffer 30 mM Tris, 1 mM EDTA, pH 8, 15 $mgmL^{-1}$ lysozyme TE buffer, sterile filter after dissolution with 0,45 µm cellulose filter), Proteinase K (>600 U/mL, recombinant, PCR grade, Thermo Fisher Scientific, Waltham, MA, USA), Lysozyme (≥90%, ≥40.000 units/mg protein, from chicken egg white, lyophilized powder, Sigma Aldrich, St. Louis, MI, USA)

Optimal volume of LAB culture: 0.5-1 mL of OD~3-2 for RNeasy Mini spin columns

Protocol 4. Before -80°C

1. Pipette 2 volumes of RNAprotect Bacteria Reagent into a 15 mL falcon tube

   The volume of the tube must be min. 4-times that of the bacterial culture
2. Add 1 volume of bacterial culture to the tube. Immediate mix by vortexing for 5 s.

   Incubation 5 min room T

3. Centrifuge 10min 5000 rpm (5417 R centrifuge, Eppendorf, Hamburg, Germany)
4. Decant the supernatant. Remove residual supernatant by gently dabbing the inverted tube once onto a paper towel.
5. Snap-freeze the pellets by liq. N_2 for -80°C storage

<u>Protocol 4</u>

1. The cell pellets are resuspended in 200 µL TE buffer with lysozyme (pipette tips up-down in falcon tube). The liquid is transferred to a sterile 2 mL eppendorf tube
2. Proteinase K 20 µL is added to the transferred liquid
3. Incubation at room T and shaking (Thermomixer comfort, Eppendorf, Hamburg Germany) for 10 min (it can be prolonged)

   Meanwhile RLT buffer is aliquoted, 2-Mercaptoethanol is added (10 $\mu L mL^{-1}$)
4. 700 µL RLT buffer+2-ME is added to the Eppendorf tube +vortex

   Centrifuge 2 min max. speed (5417 R centrifuge, Eppendorf, Hamburg, Germany)

   Use only the supernatant in the next step!
5. Add 500 µL EtOH to the Eppendorf tube (mix by pipetting)
6. Dilute RPE buffer with 4 vol. EtOH

<u>Protocol 7</u>

7. Transfer 700 µL lysate to a spin tube with 2 mL collection tube

   If the lysate is more than 700 µL, add 700 µL first, spin down first, then add more

   Centrifuge 15 s 10000 rpm (5417 R centrifuge, Eppendorf, Germany)

   Discard flow through

<u>DNase treatment</u>

8. 350 µL RW1 buffer to the spin column

Centrifuge 15 s 10000 rpm (5417 R centrifuge, Eppendorf, Germany)

Discard flow through

9. Add 10 µL DNase I stock (aliquoted in freezer) to 70 µL RDD buffer (in DNase Set). Mix gently, Centrifuge briefly to collect residual liquid if needed.
10. Add the DNase I incubation mix (80 µL) directly to the RNeasy spin column membrane (exactly on the membrane), incubate it at room T for 30-45 min
11. Add 350 µL RW1 buffer to the spin column

    Wait 5 min

    Centrifuge 15 s 10000 rpm (5417 R centrifuge, Eppendorf, Hamburg, Germany)

    Discard flow through and reuse collection tube from step 8

    <u>Repeat steps 9-10-11 once more!</u>
12. Place the spin column in a new collection tube 2 mL, add 500 µL RPE buffer to the spin column

    Centrifuge 15 s 10000 rpm (5417 R centrifuge, Eppendorf, Hamburg, Germany)

    Discard flow through

    <u>Back to Protocol 7</u>
13. Add 500 µL RPE buffer to the spin column

    Centrifuge 2 min 10000 rpm (5417 R centrifuge, Eppendorf, Hamburg, Germany)

    (no ethanol carryover is allowed, carefully remove the spin column from the collection tube so that spin column does not touch the flow through)
14. Place the spin column in a new collection tube 1.5 mL, add 30-50 µL RNase free water directly to the spin column membrane to elute RNA

Centrifuge 1 min 10000rpm (5417 R centrifuge, Eppendorf, Hamburg, Germany)

15. If the expected RNA yield is >30 µg repeat step 14 using another 30-50 µL RNase free water, or using the eluate from step 14 (>1-200 µg/mL is OK)

    Reuse collection tube from step 12

    Eluted RNA storage at -80°C

D) <u>Contamination assessment</u>

The amount of RNA (µgmL^{-1}) was measured along with the 260/280 and 260/230 absorbance ratio purity values (NanoDrop 2000, Thermo Scientific, Waltham, MA, USA). 1-2 µg of RNA samples were completed to 15 µL with RNase free water and were boiled at 75°C for 5 min in thermomixer (Thermomixer comfort, Eppendorf, Hamburg, Germany). The samples afterwards were directly put on ice for 2 min. The boiled RNA samples were spinned down for 30 s at 10000 g (5417 R centrifuge, Eppendorf, Hamburg, Germany) and 3 µL GelRed Loading dye mastermix (Biotium Inc., Fremont, CA, USA) was added to every 15 µL of boiled RNA sample. 1.5% agarose gel was prepared in 0.5x TAE buffer to run the gel with GeneRuler 1kb DNA ladder (Thermo Fisher Scientific, Waltham, MA, USA) at 80 V (PowerPac Basic system, Bio-Rad, Hercules, CA, USA) for 1 h 10 min. Pictures of the gels were taken with Geldoc (Bio-Rad, Hercules, CA, USA).

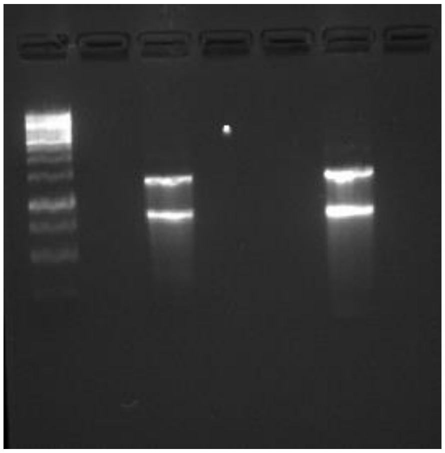

Figure S3.1. RNA gel bands, band 1 ladder, band 2-3 boiled RNA samples. Conclusions: no evidence for high load of genomic DNA contamination, only the clear bands of 23S, 16S, which corresponds to intact RNA.

E) <u>Reverse Transcription</u>

cDNA was synthesized in duplicate 20 µL reaction mixtures with 1-2 µg total RNA (RT+) or without RNA (RT-) using the High-Capacity RNA-to-cDNA™ Kit with random hexamer priming (Applied Biosystems, Thermo Scientific, Waltham, MA, USA). The reactions were carried out in duplicates in PCR tubes in thermal cycler (Mastercycler personal, Eppendorf, Hamburg, Germany) according to the kit guidelines (step 1: 37°C 60 min, step 2: 95°C 5 min, step 3: 4°C). After the synthesis, cDNA was stored at -20°C for further use.

F) qPCR Target information & qPCR oligonucleotides

For 1 target and 2 reference genes, primer pairs were designed with PrimerBLAST (https://www.ncbi.nlm.nih.gov); amplicon size, primer GC content, primer pair melting temperature difference and specificity were taken into account. The secondary structures of the amplicons were checked with “mfold” (http://unafold.rna.albany.edu/?q=mfold/RNA-Folding-Form) and their GC contents were calculated with “OligoCalc” (http://biotools.nubic.northwestern.edu/OligoCalc.html). HPLC-purified primers were obtained from Eurofins (Eurofins Genomics, Luxembourg City, Luxembourg).

Table S3.I.

Gene	Function	GENE ID	Forward primer sequence	$T_{m, Forw}$ (°C)	Reverse primer sequence	$T_{m, Rev}$ (°C)	Amplicon Size (kB)	Specificity	Amplicon GC content (%)
rluD	ribosomal large subunit pseudouridine synthase D	https://www.ncbi.nlm.nih.gov/gene/1114629	AATTCGTCCAGGAATTGTGC	56.70	TAGCAATTTCACCGTGGACA	57.45	155	OK, other product 2300	38
rpoD	RNA polymerase sigma factor RpoD	http://www.ncbi.nlm.nih.gov/gene/1114172	CATGGCGCCAGATAAAGTTC	57.24	ACCGATAGGTGTTTCGAGTGAG	59.84	73	OK	45

lacS	Lactose-galactose antiport *S. thermophylus* /note="EVIDENCE BY HOMOLOGY BIO13.04 TRANSPORT AND BINDING	*L. lactis* IL1403 complement (2062874..2063818)	TCCTTGAGCA TGTCCACCTT	58.93	AACACTTGGAACT TTCGCCC	58.97	109	OK	44

G) qPCR protocol

The qPCR mastermix for 20 µL total volume per sample with 2 µL template was the following: 1 µL cDNA sample, 1 µL 4 µM F primer (200 nM final conc.), 1 µL 4 µM R primer (200 nm final conc.), 5.7 µL water, 0.3 µL 1:500 diluted Brilliant II SYBR® Green QPCR Master Mix reference dye (ROX), 10 µL Brilliant II SYBR® Green QPCR Master Mix SYBR green (Agilent Technologies Inc., Santa Clara, CA, USA). The qPCR reactions in duplicates were performed in 96 well qPCR plates (FrameStar 96 semi skirted qPCR plates, 4titute Ltd., Wotton, UK) after spinning the well-plates down (3 min, 1500 g, Sigma 2-5 Centrifuge, Sigma GmbH, Osterode am Harz Germany) by Agilent Mx3000P qPCR System (Agilent Technologies Inc., Santa Clara, CA, USA).

Protocol for qPCR with *L. lactis* samples

1. RNA extraction & RNA quality check by gel
2. RT-step
3. qPCR

Mastermix per sample for 20 µL total volume:

- 1 µL 4 µM F primer (200 nM final conc.)
- 1 µL 4 µM R primer (200 nM final conc.)
- 0.3 µL 1:500 diluted ref. dye
- 10 µL SYBR green mix
- 2 µL template (100x dilution after cDNA synthesis step) and 5.7 µL water

Table S3.II. The qPCR thermal cycle profile.

Cycle	Duration	Temperature
**1**	3 min	95°C
**45**	20 s	95°C

	20 s	60°C
**Dissociation curve according to Agilent Mx3005P qPCR**		

H) qPCR validation

qPCR method development for bacterial retro transcribed RNA-samples for relative quantification of gene expressions.

- specificity -> melting curve analysis OK for 3 genes
- testing different primer concentrations in the mastermix -> 200 nM final primer conc.
- testing 4 different reference genes -> 2 final candidates (*rpoD, rluD*)
- inhibitor testing: serial dilutions of samples: 1x, 10x, 100x, 1000x -> 100x dilution was a compromise for inhibition and acceptable range of C_q
- contamination assessment:

  NTC is ok if C_q difference is >10 (pass in all cases)

  RT- is ok if C_q difference is > 4-5 (bacterial samples, sufficient difference, else “NOT OK”)
- primer efficiency is ok in the range 80-110% (evaluation of the primer design – all primer pairs passed in the dilution range 50x-1000x)

Table S3.III. Primer efficiencies

gene	Efficiency %	R^2
lacS	100	0.99
rpoD	104	0.99
rluD	91	0.98

I) Data analysis

The qPCR data was evaluated with the MxPro software (Agilent Technologies Inc., Santa Clara, CA, USA) and Excel.

- C_q determination: threshold was manually set at the take-off point of the amplification curves in order to align the thresholds of multiple plate qPCR study
- Possible outliers in terms of the shape of amplification curves were removed.
- Choice of 2 ref. genes: similar and constant expression pattern throughout the samples, similar relative gene expression results with both reference genes for the target genes.

For calculations of relative gene expressions, 2 methods were used:

- the so called ΔΔCq equation when primer efficiency is sufficient

  $$-\Delta\Delta Cq = -(\Delta Cq1 - \Delta Cq2)$$

  $$\frac{SampleA}{SampleB} = 2^{-\Delta\Delta Cq}$$

  $$\Delta Cq1 = CqAtarget - CqAref$$

  $$\Delta Cq2 = CqBtarget - CqBref$$

- calculations with real primer efficiencies

  $$\frac{SampleA}{SampleB} = \frac{(1 + E_{ref})^{CqAref - CqBref}}{(1 + E_{tar})^{CqAtar - CqBtar}}$$

Repeatability

- Multiple plate qPCR study -> inter-plate calibrator sample was used and C_q values remained consistent, standard deviation randomly distributed among plates (no trend, no drift)

Statistical methods for results significance

The expression fold-difference between aerobic and respiration-permissive (aerobic hemin-supplemented) galactose batch samples was tested with a one-tailed t-test at a 95% confidence interval (alpha 0.05): Are the

target gene expressions of samples taken from aerobic cultures the same with gene expressions of samples taken from respiration-permissive cultures?

H_0: $\mu_D=0$ (yes/accept, they are the same, there is no significant difference between the gene expressions)

H_A: $\mu_D \neq 0$ (no/reject, they are not the same, there is a significant difference between the gene expressions)

References

1. Joubert L, Derre-Bobillot A, Gaudu P, Gruss A, Lechardeur D (2014) HrtBA and menaquinones control haem homeostasis in *Lactococcus lactis*. Mol Microbiol 93:823-833. doi:10.1111/mmi.12705

2. Oliveira AP, Nielsen J, Förster J (2005) Modeling *Lactococcus lactis* using a genome-scale flux model. BMC Microbiology 5:1-15. doi:10.1186/1471-2180-5-39
